# Evaluation of a real-time virtual intervention to empower persons living with HIV to use therapy self-management: study protocol for an online randomized controlled trial

**DOI:** 10.1186/1745-6215-13-187

**Published:** 2012-10-05

**Authors:** José Côté, Gaston Godin, Yann-Gaël Guéhéneuc, Geneviève Rouleau, Pilar Ramirez-Garcìa, Joanne Otis, Cécile Tremblay, Ghayas Fadel

**Affiliations:** 1 Research Chair in Innovative Nursing Practices, Research Centre of the Centre Hospitalier de l’Université de Montréal, Quebec, Canada; 2 Université de Montréal, Quebec, Canada; 3 Canada Research Chair on Behaviour and Health, Université Laval, Quebec, Canada; 4 Canada Research Chair on Software Patterns and Patterns of Software, École Polytechnique Montreal, Quebec, Canada; 5 Canada Research Chair in Health Education, Université du Québec à Montréal, Quebec, Canada; 6 Université de Montréal, Quebec, Canada; 7 Research Centre of the Centre Hospitalier de l’Université de Montréal, Quebec, Canada; 8 Quebec Coalition Of Community-Based HIV/AIDS Organizations (COCQ-SIDA), Montreal, Quebec, Canada

**Keywords:** Randomized controlled trial, Web-based study, People living with HIV, Self-management program, Adherence, Anti-retroviral therapy

## Abstract

**Background:**

Living with HIV makes considerable demands on a person in terms of self-management, especially as regards adherence to treatment and coping with adverse side-effects. The online HIV Treatment, Virtual Nursing Assistance and Education (Virus de I’immunodéficience Humaine–Traitement Assistance Virtuelle Infirmière et Enseignement; VIH-TAVIE™) intervention was developed to provide persons living with HIV (PLHIV) with personalized follow-up and real-time support in managing their medication intake on a daily basis. An online randomized controlled trial (RCT) will be conducted to evaluate the efficacy of this intervention primarily in optimizing adherence to combination anti-retroviral therapy (ART) among PLHIV.

**Methods/design:**

A convenience sample of 232 PLHIV will be split evenly and randomly between an experimental group that will use the web application, and a control group that will be handed a list of websites of interest. Participants must be aged 18 years or older, have been on ART for at least 6 months, and have internet access. The intervention is composed of four interactive computer sessions of 20 to 30 minutes hosted by a virtual nurse who engages the PLHIV in a skills-learning process aimed at improving self-management of medication intake. Adherence constitutes the principal outcome, and is defined as the intake of at least 95% of the prescribed tablets. The following intermediary measures will be assessed: self-efficacy and attitude towards antiretroviral medication, symptom-related discomfort, and emotional support. There will be three measurement times: baseline (T0), after 3 months (T3) and 6 months (T6) of baseline measurement. The principal analyses will focus on comparing the two groups in terms of treatment adherence at the end of follow-up at T6. An intention-to-treat (ITT) analysis will be carried out to evaluate the true value of the intervention in a real context.

**Discussion:**

Carrying out this online RCT poses various challenges in terms of recruitment, ethics, and data collection, including participant follow-up over an extended period. Collaboration between researchers from clinical disciplines (nursing, medicine), and experts in behavioral sciences information technology and media will be crucial to the development of innovative solutions to supplying and delivering health services.

**Trial registration:**

CE 11.184 / NCT 01510340

## Background

The morbidity and mortality rates of people infected with HIV has declined dramatically since the advent in 1996 of combination antiretroviral therapy (ART) and the use of appropriate prophylaxis against opportunistic infections [1,2]. Formerly classified as a terminal illness, HIV infection is today considered a chronic disease. Although ART cannot eradicate HIV, existing antiviral therapies suppress viral replication and thus make it possible to maintain and improve the immune function of infected individuals. However, adherence to treatment is key to preventing treatment failure and the development of antiviral drug resistance.

A certain degree of non-adherence might have an effect on treatment outcome, depending on pattern and medication. For example, individuals who skip treatment on consecutive days (sustained interruption) are at higher risk of presenting a detectable viral load than are those who skip an equal number of doses non-consecutively (interspersed missed doses) [3,4]. Furthermore, it has been recognized that, because of their long half-lives and potency, certain types of medication, such as non-nucleoside reverse-transcriptase inhibitors (NNRTIs) and boosted protease inhibitors (PIs), are more forgiving of moderate levels of adherence [5,6]. Notwithstanding, poor adherence remains a major risk factor in virologic failure and the development of resistance [7]. Therefore, developing interventions to boost and sustain ART adherence has become a crucial objective in the field of HIV management.

### Interventions to foster adherence

Treatment adherence immediately arose as a key issue after the introduction of ART. The need to develop appropriate support interventions to foster adherence was crucial at the time, and it remains strong today, despite the fact that regimens have grown simpler [8] (smaller doses, fewer pills) and easier to tolerate over time. After all, ART constitutes a lifetime commitment on the part of the infected individual. Since 2003, the results of pilot studies have demonstrated the feasibility of interventions to influence adherence [9-12], and some interventions evaluated in experimental studies have shown a potential to optimize adherence [13-22]. A systematic review of treatment adherence interventions intended for persons living with HIV (PLHIV) [23] suggested that individual interventions targeting medication management skills were associated with improved adherence outcomes (*n* = 10 studies). In their systematic review of interventions intended to optimize medication adherence among other groups, Haynes *et al*. [24] indicated that only 36 of the 83 interventions examined were associated with improved adherence. Kripalani *et al.*[25] reported similar results in their review focused on a chronically ill group. Of the 37 randomized trials reviewed, 20 produced a significant change in at least one adherence marker, and over half of these interventions (n = 11) had an effect on clinical markers.

In light of these mixed results, Haynes *et al*. [24] stressed the need for novel approaches to support treatment adherence. Recent reviews by Bärnighausen *et al*. [26] and Horvarth *et al*. [27] have suggested that interventions assisted by information and communication technologies, such as text messages sent via mobile phone, could effectively increase adherence in sub-Saharan Africa. Thus, tailored interventions and the use of information technologies open up interesting possibilities for developing innovative interventions geared to optimizing treatment adherence and reaching a broader patient base across vast geographical areas.

### Information and communications technologies: a promising avenue for providing support to people living with HIV

In the field of health education, there is a growing and apparently inevitable tendency to use information and communication technology (ICT), such as web applications and software systems, as interactive means of information transmission [28-31]. These technologies make it possible not only to present a large amount of information in a more user-friendly way, but also for users to access, at their convenience, information better adapted to their own needs. This characteristic greatly facilitates the active learning process at the heart of the development and reinforcement of health behavior self-management skills [32-34].

Tailored interventions are defined as change strategies intended for a given person based on their specific characteristics, identified beforehand through an individual evaluation [35,36]. What distinguishes these interventions is the fact that the change strategy targets the needs of one particular person rather than those of a group, and that the messages and feedback conveyed are adapted to individual factors related to a given health behavior [37]. This makes it possible to achieve more significant results as the person feels more engaged by the messages and in turn engages more readily in the proposed strategies [38-40].

The combined benefits of tailored interventions and of the use of ICT make this type of intervention more effective than traditional health education interventions [34]. Studies have confirmed the efficacy of this type of intervention on the adoption of various health behaviors, including quitting smoking [41-45], reducing the amount of fat in the diet [46,47], increasing the consumption of fruit and vegetables [48,49], and engaging in early cancer detection [50,51]. In the field of HIV, ICT has been used to encourage the adoption of safe behaviors whether in terms of sexual practices or use of syringes [52-56].

In their review of computer-assisted tailored interventions delivered online, Lustria and colleagues [57] identified 30 studies (out of the 503 found between 1996 and 2007) where the applications targeted four health behaviors (nutrition (*n* = 10), physical activity (*n* = 7), tobacco use (*n* = 7), alcohol consumption (*n* = 3)) , as well as ‘other behavior’ (*n* = 3). In their analysis, they sought in particular to describe the mechanisms used to put into operation the concept of tailored interventions and to implement online interventions. They found a wide variety of approaches being used, and highlighted the importance of pursuing research in this domain.

The systematic review (*n* = 24 studies) by Murray *et al*. [58] supported the effects of computer-assisted interventions for individuals living with a chronic condition in terms of knowledge acquisition, changes to health behavior, self-efficacy, perceived social support, and other clinical markers. These technologies have been used for individuals living with asthma [59], diabetes [60-62], heart disease [63-66], and HIV [67,68]. On the whole, what emerges is that these technologies can be used with a variety of groups of different ages [69-73]. The use of different ICTs in the field of health education shows that they are effective in transferring knowledge and developing the skills needed for health promotion and management of the demands inherent to illness [29,30].

Regarding access to these technologies, 175 million adults [74] in the USA reportedly used the internet in 2010 to obtain health-related information. According to the results of a Harris poll, 88% of adults online have searched the web for such information. Moreover, in 2009, Statistics Canada [75] reported that 80% of Canadians 16 years of age or over used the internet at home for personal reasons, and this percentage represented 21.7 million users. These Canadian data also showed that 70% of people used the internet to search for medical information and for health-related purposes (74% of women and 66% of men). In Quebec, the second most densely populated province in Canada, the NETendances survey conducted by CEFRIO-Léger Marketing [76] showed that 79.1% of *Québécois* had used the internet in the previous 7 days.

To date, web applications have been developed to support primary and secondary prevention efforts for tobacco use, physical activity, nutrition, use of certain health examinations, and the adoption of safe sexual behaviors. However, few tailored interventions have used this educational approach in tertiary prevention to support treatment adherence. Living with HIV makes considerable demands on a person in terms of self-management, especially regarding adherence to lifelong therapy and coping with adverse side-effects. PLHIV must play a more active role in this management, thus breaking with the traditional paternalistic health model in which the patient has a more passive role. Instead, the health professional becomes a care partner and mentor who empowers the PLHIV to take charge of the situation. Consequently, it is imperative that innovative ways should be found to provide assistance and follow-up to PLHIV. From this point of view, we believe that the use of ICTs can constitute an effective approach to treatment management by providing PLHIV with support in the form of messages that target and are adapted to their needs. Such virtual follow-up would be complementary to existing clinical follow-up. Given the persistent or long-term nature of ART, finding new ways to support PLHIV who are on such treatment seems an avenue worth exploring.

### Goal

Drawing on the recommendations of the National Institutes of Health (NIH) [77] regarding the four phases of research on interventions in the field of health sciences, we plan to evaluate the efficacy of an online virtual intervention in optimizing adherence to antiretroviral medication intake by PLHIV (phase III). The study will rest on preliminary work (phases I and II) that served to clearly identify the predictors of treatment adherence by PLHIV [78], and will develop and validate the content of the intervention in question [79], and create the corresponding web application [80].

### Preliminary work

Based on a philosophy of empowerment, the HIV Treatment, Virtual Nursing Assistance and Education (Virus de l’immunodéficience Humaine–Traitement Assistance Virtuelle Infirmière et Enseignement; VIH-TAVIE™^a^) web application makes it possible to develop and strengthen the skills needed for PLHIV to self-manage their treatment while boosting their sense of self-efficacy with respect to the daily intake of medication [80]. This application was developed in accordance with the systematic analytic process proposed by Bartholomew *et al.*[81,82], and rests on explanatory and predictive models of health behaviors [79]. The specificity of this application lies in its action mechanism based on Bandura’s theory of behavioral change [83], which targets the development and reinforcement of skills, that is, the individual’s ability to act.

The web application is composed of four interactive computer sessions hosted by a virtual nurse who engages the PLHIV in a skills-learning process aimed at improving self-management of medication intake. The skills covered (motivational, self-awareness, problem-solving, emotion-regulation, social) allow the PLHIV to integrate the therapeutic regimen in their daily routine, manage adverse side-effects, handle problem situations that might interfere with medication intake, interact with health professionals, and mobilize their social support network. Aside from delivering tailored teaching, the virtual nurse refers to the experiences of other individuals who coped with similar situations successfully. During these sessions, the virtual nurse provides feedback and positive reinforcement on the individual’s personal style and methods and on the acquired skills. The interactive system is designed to allow repeated applications and backtracking depending on the individual’s needs. Moreover, at each session, feedback is provided on the significant elements of the previous session. The individual’s profile is personalized as a function of their needs and characteristics, thus giving the profile a tailored fit. Furthermore, the computer innovation underlying the virtual intervention allows to create new pages , thus allowing the system administrator the flexibility to add or modify page models and content depending on the context.

## Methods

### Ethics approval

The study has been approved by the Research Ethics Board of the Université de Montréal and the Research Center of the Centre Hospitalier de l’Université de Montréal.

## Design

We opted for an online randomized controlled trial (RCT) to evaluate the efficacy of the virtual intervention (web application) primarily in influencing ART adherence. Adherence is a behavioral indicator that can be predicted in part by cognitive and affective variables, particularly sense of self-efficacy and attitude towards antiretroviral medication intake [78]. These variables are in turn explained by perceived social support, relationship with health professionals, and absence of adverse effects. These intermediate variables, which are the targets of our intervention, will allow effective change in the short term. They constitute mediators capable of explaining the intervention’s effect on adherence. There will be three measurement times: baseline (T0), after 3 (T3) and 6 months (T6) of baseline measurement. Participants will be drawn from a convenience sample and will be randomly assigned either to an experimental group that will use our web application, or to a control group that will be presented with a list of various websites of interest.

### Hypotheses

The primary hypothesis is that a higher proportion of participants in the experimental group will prove treatment-adherent at T6 compared with the control group. The explanatory hypothesis is that the following variables are mediators that can explain the intervention’s effect on adherence: sense of self-efficacy, attitude towards antiretroviral medication intake, degree of symptom-related discomfort, and perceived social support (T0, T3, T6).

### Sample

We aim to recruit a sample of 232 participants, that is, 116 per group. The PLHIV who will participate in the study must be at least 18 years of age and on ART for at least 6 months. The reason for the latter criterion is that we wish the therapy-management intervention to focus on adherence over time rather than on treatment initiation. Internet access is another inclusion criterion. This will introduce a selection bias in the study; however, the bias will be offset by the inherent value in evaluating an innovative service-delivery method that, in the near future, could reach a vast number of people at low cost and, thus, constitute an important option for providing follow-up to groups with a chronic health problem. In this study, both the experimental group and the control group will be composed of PLHIV with similar characteristics, who have internet access and who use it to obtain information relative to their health condition.

The sample size was estimated based on the studies by Tuldrà *et al.*[84] and Pradier *et al*. [18] carried out with PLHIV, and on the systematic review by Haynes *et al*. [24] of adherence-related interventions intended for various groups. In the study by Tuldrà *et al*. [84], there was a difference of 25 percentage points between the control group and the experimental group in terms of treatment adherence (69% vs. 94%, respectively). For Pradier *et al.*[18], the proportion of treatment-adherent participants was 58% in the experimental group and 63% in the control group at the start of the study, and 75% and 60% respectively after the intervention. We calculated our sample size on the assumption that the proposed intervention (the web application) will increase the percentage of treatment-adherent participants in the experimental group by 20%. The percentage of treatment-adherent participants at the start of the study will be the same as that reported by Godin *et al*. [78] (that is, 50%), given that our recruitment pool is very similar to theirs. This is a conservative assumption given that the variance in the difference between the two proportions will be larger, with the benchmark proportion set at about 50%. Consequently, the sample size required to detect a difference of 20% (50% treatment-adherent participants in the control group versus 70% in the experimental group) will be larger than for other scenarios with the same 20-point difference. Hence, to detect a difference of 20 percentage points at 80% power and a χ^2^ test two-tailed α value of 0.05, the required sample size is 186 participants, that is, 93 per group. As we are assuming a 20% attrition rate (that is, the same rate seen in the studies by Goujard *et al*. [14] and Tuldrà *et al.*[84]), the sample size has been set at 232 participants. With a sample of 186 participants completing the study, the statistical power will be enough to detect a moderate effect on the intermediate variables. In particular, with *n* = 186, an α value of 0.05 (two-tailed Student’s *t*-test) will allow detection of the difference between the two percentages, corresponding to a standard deviation of 50% and power of greater than 90%.

### Experimental conditions

The virtual intervention consists of four computer sessions of 20 to 30 minutes in length. There will be a 1-week interval between sessions to ensure the progressive acquisition and consolidation of skills, thus, access will be controlled and pre-determined within this interval. It will be possible at all times for participants to revisit previous sessions. Access will be unlimited in terms of intensity, frequency, and time of use throughout the duration of the study. Delivering an intervention by computer affords a key advantage in that it allows complete control of the content. Moreover, the intervention’s parameters are recorded, thus making it possible to obtain a reliable picture of the actual intensity, duration, and frequency of the intervention. These data will be available for subsequent analyses.

### Control group

Participants in the control group will be invited to consult, at their convenience and from the location of their choice, a list of pre-determined websites offering information on antiretroviral medications, their side-effects, and their interactions.

### Measurement

Adherence will constitute the principal outcome, and for the purposes of the study, it will be evaluated through a self-administered questionnaire that was developed for and validated on the target population in accordance with recommendations relative to the measure of treatment adherence [85]. The questionnaire comprises seven items that serve to determine how many times a person forgets to take their medication. The questionnaire is designed to place the respondent in a context where events and situations could lead to lapses. The questionnaire’s validity was assessed (sensitivity 71%; specificity 72%; correct classification 72%; odds ratio 6.15) using immunologic (CD4 count) and virologic (viral load) parameters as validation criteria. Adherence was defined as the intake of at least 95% of the prescribed tablets. Although there is no clear minimum cut-off point defining what constitutes sufficient ART adherence for achieving optimal treatment efficiency [5], this is generally set at between 90% and 95% [86]. Self-reported viral load and CD4 counts will also be recorded.

The intermediary measures will be assessed by means of validated instruments. Self-efficacy regarding medication intake will be measured using 12 items rated on a 5-point Likert scale (here is an example of an item: “I can take my antiretroviral medications even if I’m in the company of people who don’t know I’m seropositive”). These items are adapted from a scale developed by Godin *et al*. [78] and have previously been used on a large sample (*n* = 399). To adapt the instrument to our context (that is, antiretroviral medication intake), the items have been reformulated by expert consensus following the results of a focus group and of a literature review, and on the basis of Bandura’s theoretical model of self-efficacy. A content validation has also been carried out.

Attitude toward medication intake will be evaluated through six items rated on a five-point Likert scale. These items emerged from focus groups of PLHIV who were on treatment. A preliminary version of the instrument was tested on 35 PLHIV. The scale was previously used on a large sample (*n* = 399) [78], obtaining a Cronbach’s α coefficient of 0.83 and a test-retest reliability coefficient of 0.72.

Symptom-related discomfort will be measured using the Self-Completed HIV Symptom Index [87]. This 20-item instrument determines using a scale of 1 to 4 whether symptoms are present (0 = absence) and the degree of discomfort experienced. The instrument has been validated on 188 PLHIV, and it was found to have acceptable psychometric properties, including good construct validity.

Social support will be evaluated using the Medical Outcome Survey [88,89]. One dimension of social support will be measured by subscale of emotional support, which has eight item, each of which is rated on a five-point Likert scale. The instrument has been found to have good content validity and appreciable internal consistency.

A sociodemographic questionnaire will cover personal characteristics such as sex, age, family situation, level of education, employment situation, number of children, and questions about HIV, the therapeutic regimen, and presence of symptoms.

### Recruitment and online data collection

The study will be conducted entirely online. The project will be advertised on the websites of resources available to PLHIV, where a hyperlink and a banner will be inserted to redirect parties interested in participating in the online research. The targeted resources include partner clinics, community agencies, and other organizations that deliver teaching, care, treatment, or follow-up to PLHIV. In addition, health professionals and stakeholders will be invited to inform their PLHIV about the study. To promote the project among PLHIV, different communication strategies will be used: 1) traditional methods, such as poster and information brochures; and 2) technological or virtual methods, by advertising the study on websites of organizations (medical clinics, association for PLHIV) that are often visited by PLHIV.

Individuals who will visit the study’s website will view a short video clip of testimonials by a few PLHIV, then they will be given information on the study. After accepting the conditions of the study and consenting to take part in it, they will sign up for the study by providing an email address and a pseudonym. Each participant will be validated through an email address check. Once this step is completed, a hyperlink will be emailed to allow participants to access the first questionnaire online on the website, and only after having completing this questionnaire will they be randomly assigned by the computer system to the experimental group or the control group. Participants in the control group will be invited to consult pre-determined websites that offer information on ART and its associated side-effects. Participants in the experimental group will be offered the virtual intervention through the web application. At 3 and 6 months after the initial measurement, participants will complete the online questionnaires again. A reminder to this effect will be emailed to them in the days prior to the scheduled measurement. Participants will be compensated for their time spent on the study with a gift certificate for each measurement time completed.

### Type of analysis used

To establish whether the experimental and control groups are equivalent, the means and medians of the continuous variables and the frequency distributions of the discrete variables will be compared using descriptive analyses. The principal analyses will focus on comparing the two groups in terms of treatment adherence (the study’s main outcome, considered as a binary variable with adherent being ≥ 95% and non-adherent > 95%) at the end of follow-up at T6. An intention-to-treat (ITT) analysis will be carried out to evaluate the true value of the intervention in a real context. Consequently, in the following analyses, all participants will be assessed in their respective randomization group as to whether they are following the procedure or have been lost to attrition during follow-up. In accordance with the principles of ITT, participants who drop out and those for whom there is no adherence measure at T6 will be considered as non-adherent.

The primary hypothesis is that the VIH-TAVIE intervention will increase the proportion of treatment-adherent patients at T6. This hypothesis will be tested using a χ^2^ test with 1 degree of freedom. If preliminary analyses show that the two groups differ on certain characteristics, multivariate logistic regression will be used to adjust the intervention’s effect on these characteristics, which will be considered as potential confounding variables. In this case, the two-tailed Wald’s test will be used to test the intervention’s adjusted effect.

For the secondary analyses, the two groups will be compared on all the repeated measures of adherence (T3 to T6) using a generalized estimating equation (GEE) approach that generalizes the logistic regression for longitudinal data with repeated measures of the binary measure [90]. We will assume that the covariance matrix has an auto-regressive structure of order = 1 to account for inter-correlations between repeated measures (over the course of follow-up) with the same participants. The difference between repeated measures of adherence (T3 and T6) will constitute the binary dependent variable. The binary intervention indicator, the indicator of time since the follow-ups (3 and 6 months) and, if necessary, the confounding variables, will be included as independent variables. Finally, the interaction between the intervention effect and the time effect will be added to the GEE model and will be tested to verify whether the intervention’s effect on adherence varies over time. In the GEE analyses, a conservative approach will be used and, accordingly, all missing data for the dependent variable (adherence) will be replaced with ‘non-adherent’. The same approach will be used to evaluate the effects of the intervention on the repeated measures of the intermediary variables. However, given that these variables will be measured by means of quantitative scales, a linear mixed model for repeated measures of a continuous dependent variable [91] will be used instead of the GEE model.

## Discussion

### Challenges of an online randomized controlled trial

Carrying out this online RCT poses various challenges, particularly in terms of recruitment, ethics, and data collection, including participant follow-up over an extended period [92].

First, to attract and retain the online participation of PLHIV interested in the study, the team sought the help of web design experts. This help led to the development of a brand image (concept of branding) by an artistic director, who created a logo that would carry the image of the study and be easily recognizable by our target group. This branding set the tone (in terms of color and visuals) for our website pages, which were created to be visually attractive to participants. More specifically, upon reaching the first page of the website, participants will listen to testimonials by a few PLHIV about their own experiences with medication intake and with browsing the web in search of health-related information. Aside from being an interesting and dynamic visual tool, testimonials have been used to allow potential participants to identify with the intervention, and to humanize and personify the web-based study. These testimonials also have the potential to strengthen the branding of the intervention. Then, in a short video clip, the research coordinator and a project spokesperson will present information on the study, including its aim and inclusion criteria, and explain what participation entails.

Various communication strategies will be used to promote the study among PLHIV. First, traditional methods of dissemination will be used, such as publishing advertisements or short articles in magazines aimed at PLHIV, and distributing information brochures and posters to the clinics and community organizations targeted. Second, an internet-based approach will also be used, consisting of posting a hyperlink to our study on the websites of organizations (medical clinics, association for PLHIV, community groups) frequently visited by PLHIV. In this regard, Birnbaum [93] suggested that such organizations should run advertisements in their electronic newsletters. A list of targeted internet media will be compiled in collaboration with key informants working with PLHIV in community organizations. There are other methods of online recruitment, such as reaching target groups through chatrooms, sending e-mails to pre-established lists of participants, or posting banners on popular websites [94,95]; however, we will not use these latter strategies in our study. According to Noseck *et al*. [95], even if posting advertising banners is a popular way of promoting a study, it remains a costly means of recruitment and ‘is not the most effective at netting traffic’ ([95], p. 167).

One of the risks that remain difficult to control or prevent in a virtual environment is having the same person participate more than once in a given study [93,95,96]. Although the incidence of this happening has been shown to be small, at 3%, [93,97], certain strategies can be used to detect or prevent it and reduce the risk of its occurrence, including warning participants to register only once, asking for an email address and a password at registration, and filtering data that seem to be identical[93,95,96].

The ethical aspects of online studies have been discussed by various authors [98-101]. The topic has received special attention because of the online research environment in itself, where there’s no direct interaction between researchers and participants [95,96]. Among other things, consent to participate can be presented in the form of a statement to be read and checked off before clicking on a button labeled ‘I accept’. A button labeled ‘withdraw’ or ‘quit the study’ [95] will also be accessible to allow participants the freedom to drop out of the study at any time. As proposed by Hewson [99] and Nosek *et al*. [95], a ‘debriefing page’ and contact information for a resource person in the event of questions or problems will also be provided. The information must be presented in such a way as to properly inform participants but without overwhelming them with data, which could cause difficulties and discourage subjects from participating.

The security and confidentiality of electronic data in online studies are serious concerns that necessitate the implementation of stringent measures. The data collected through the online questionnaires will be stored on a secure server located at the research center where the research work is carried out. Only system administrators will have access to the data on the server. To protect the identity of participants, only an email address and a pseudonym will be requested from participants, as this information is necessary to ensure follow-up and disseminate results, as pointed out by Michalak and Szabo [100]. Using a firewall, encrypting the data, protecting passwords, and separating the sociodemographic data from the experimental data (linking them through a code) reduces the risk of an unauthorized third party accessing confidential content [95,101]. A database has been planned to support the subsequent direct transfer of the data to analysis software (the SPSS package; SPSS Inc., Chicago, IL, USA) in order to facilitate data analysis.

As emphasized by Bull *et al*. [94], online longitudinal evaluations entail following up participants and completing various measures. According to Bull *et al.*[94] and Bennett and Glasgow [92], online studies have a lower retention rate than that of traditional studies, and attrition rates of 40% are not uncommon. However, Schubart *et al.*[102] and Bull *et al*. [103] highlighted a number of elements that foster a high retention rate in online studies, including the availability of a health professional to provide feedback, the dynamic qualities of the study website, and the inclusion of a clinician coach, such as a nurse, in the intervention. All of these will be considered in this study.

For the purpose of encouraging participants to continue with the study, reminder e-mails (maximum of three to four) will be sent out at 7-day intervals prior to measurement. Bull and colleagues [94] noted that e-mails alone were not sufficient to communicate with participants, and that a variety of communication modes should be used. They suggested that participants should be asked to provide other contact details such as home and mobile telephone numbers, and a home address. According to these authors, a balance must be struck between guaranteeing confidentiality and ensuring follow-up. Because the technology we plan to use is complex and can present many problems and bugs, we plan to assign a resource person (technical support) to help users in this regard. According to Paul and colleagues [101], this constitutes a key source of support if the research budget allows it.

A research project that unfolds entirely online necessitates that all parties share an understanding of the specific terminology used by the various experts involved and of the role played by the professionals in each of the project’s teams (computer programming, multimedia, clinical). It is necessary to ‘think virtual’, that is, to make sure that procedures applied in the real world are operational online. Transposing research procedures to an entirely virtual research intervention entails anticipating the entire sequence of participation and follow-up of the target persons. Making this shift is not easy. Indeed, the absence of a common language between research, clinical, and web media teams, as well as a mutual misunderstanding of the needs of each group (researchers versus tech support teams, for instance), calls for a great deal of clarification and numerous meetings to make the technical operations required by the project possible. This collaboration between researchers from clinical disciplines (nursing and medicine), experts in behavioral and educational sciences, and information technology and media experts is crucial to the development of innovative solutions to supplying and delivering services to those who need them.

However, without the contribution of community partners and other clinical collaborators, such a study could not be carried out easily. Their support and expertise are invaluable at all levels, particularly in planning and developing the different stages of the online study (for example, using testimonials was our partners’ idea). Our partners will also be involved in promoting the study, recruiting participants, and ultimately transferring and implementing the virtual intervention.

## Trial status

Data collection was started in February 2012.

## Endnote

^a^The acronym VIH-TAVIE (Virus de l’immunodéficience Humaine–Traitement Assistance Virtuelle Infirmière et Enseignement) contains a wordplay in French that can be rendered in English as ‘live your life’.

## Competing interests

The authors declare that they have no competing interests.

## Authors’ contributions

JC and GG drafted the study protocol. JC and GR adapted the protocol for the publication. All authors critically revised and approved the final manuscript.
